# Development of an improved and specific inhibitor of NADPH oxidase 2 to treat traumatic brain injury

**DOI:** 10.1016/j.redox.2023.102611

**Published:** 2023-01-18

**Authors:** Hannah Mason, Ganesha Rai, Arina Kozyr, Nathaniel De Jonge, Emily Gliniewicz, Lars J. Berg, Gal Wald, Cayce Dorrier, Mark J. Henderson, Alexey Zakharov, Tristan Dyson, John Audley, Anthony M. Pettinato, Elias Carvalho Padilha, Pranav Shah, Xin Xu, Thomas L. Leto, Anton Simeonov, Kol A. Zarember, Dorian B. McGavern, John I. Gallin

**Affiliations:** a Viral Immunology and Intravital Imaging Section, National Institute of Neurological Disorders and Stroke, National Institutes of Health, Bethesda, MD, 20892, USA; b Clinical Pathophysiology Section, Laboratory of Clinical Immunology and Microbiology, National Institute for Allergy and Infectious Diseases, Bethesda, MD, 20892, USA; c National Center for Advancing Translational Sciences, National Institutes of Health, Rockville, MD, 20850, USA; d Molecular Defenses Section, Laboratory of Clinical Immunology and Microbiology, National Institute for Allergy and Infectious Diseases, Bethesda, MD, 20892, USA

**Keywords:** NADPH Oxidase, Traumatic brain injury, Inhibitor, Mouse

## Abstract

NADPH oxidases (NOX's), and the reactive oxygen species (ROS) they produce, play an important role in host defense, thyroid hormone synthesis, apoptosis, gene regulation, angiogenesis and other processes. However, overproduction of ROS by these enzymes is associated with cardiovascular disease, fibrosis, traumatic brain injury (TBI) and other diseases. Structural similarities between NOX's have complicated development of specific inhibitors. Here, we report development of NCATS-SM7270, a small molecule optimized from GSK2795039, that inhibited NOX2 in primary human and mouse granulocytes. NCATS-SM7270 specifically inhibited NOX2 and had reduced inhibitory activity against xanthine oxidase *in vitro*. We also studied the role of several NOX isoforms during mild TBI (mTBI) and demonstrated that NOX2 and, to a lesser extent, NOX1 deficient mice are protected from mTBI pathology, whereas injury is exacerbated in NOX4 knockouts. Given the pathogenic role played by NOX2 in mTBI, we treated mice transcranially with NCATS-SM7270 after injury and revealed a dose-dependent reduction in mTBI induced cortical cell death. This inhibitor also partially reversed cortical damage observed in NOX4 deficient mice following mTBI. These data demonstrate that NCATS-SM7270 is an improved and specific inhibitor of NOX2 capable of protecting mice from NOX2-dependent cell death associated with mTBI.

## Highlights

• A new NOX2 inhibitor, NCATS-SM7270, protects mice from mild traumatic brain injury.
• Knockout of NOX1 or NOX2 protects from injury while deletion of NOX4 increases it.
• NCATS-SM7270 partially reversed mTBI in NOX4KO mice.

## Graphical abstract

**Figure:**
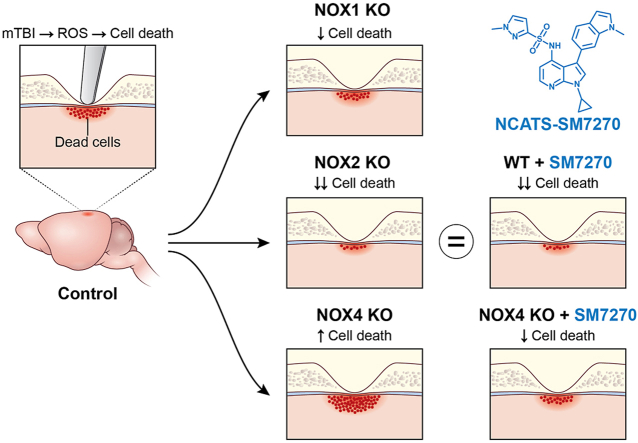

## 1 Introduction

Production of ROS by NOX2 in inflammatory cells is an essential part of innate host defenses [1], but its absence can protect from inflammatory diseases such as atherosclerosis [2,3] and TBI [4], suggesting that NOX2 may be an attractive druggable target for these diseases [5]. Pharmacologic inhibition of NOX proteins and ROS has been pursued for the treatment of cancer [6], hepatic and pulmonary fibrosis [7], Type 1 Diabetes [8], and TBI [[9], [10], [11], [12], [13], [14]]. Myelomonocytic cells recruited to brain lesions following TBI play a significant role in producing ROS [14,15], and NOX's were shown to be increased in tissues of the central nervous system (CNS) after head injury [[11], [12], [13]]. In the CNS, NOX1, NOX2, and NOX4 predominate and are expressed in various resident cells including neurons, astrocytes, and microglia [[16], [17], [18], [19]]. Genetic ablation of NOX2 in moderate-to-severe TBI models has repeatedly shown NOX2 knockout (KO) mice to be resistant to head injury, with reduced lesion volumes and improved behavioral scores [18,20,21]. Other knockout studies have suggested that NOX4 is beneficial [22]. However, no studies to date have tested knockouts of the different NOX isoforms using the same TBI model.

Due to the pathophysiologic roles of ROS in TBI, attempts have been made to utilize non-specific ROS scavengers (e.g, glutathione, apocynin) for protection as well as non-specific compounds that inhibit the production of ROS by NADPH oxidases as a class (e.g., gliotoxin and diphenyleneiodonium), but these molecules also act on other important cellular systems [[9], [10], [11], [12],[23], [24], [25]]. Much work has, therefore, been devoted to the development of more specific inhibitors of NOX isoforms. This goal has remained elusive, however, because NOX and dual oxidase (DUOX) enzymes are related, sharing structural identities, regulatory partners, and biochemical cofactors.

GSK2795039, a putative NOX2 inhibitor, was shown previously to reduce inflammation [26] and TBI pathology in mice [27]. However, the short half-life, lack of selectivity of this compound [26] and the discovery of a large number of metabolites [28] suggested that structural modifications may improve stability. Here, we describe 25 related analogs of GSK2795039, some of which have improved NOX2 inhibitory activity, isoform specificity, and pharmacokinetic properties. We focused our attention on NCATS-SM7270 which we show has greatly improved inhibitory specificity for NOX2 relative to other NOX family members, comparable activity in human and mouse leukocytes, and protective activity in a mouse model of mTBI.

## 2 Results

### 2.1 Identification of small molecule NOX2 inhibitors with improved specificity

**Fig. 1:**
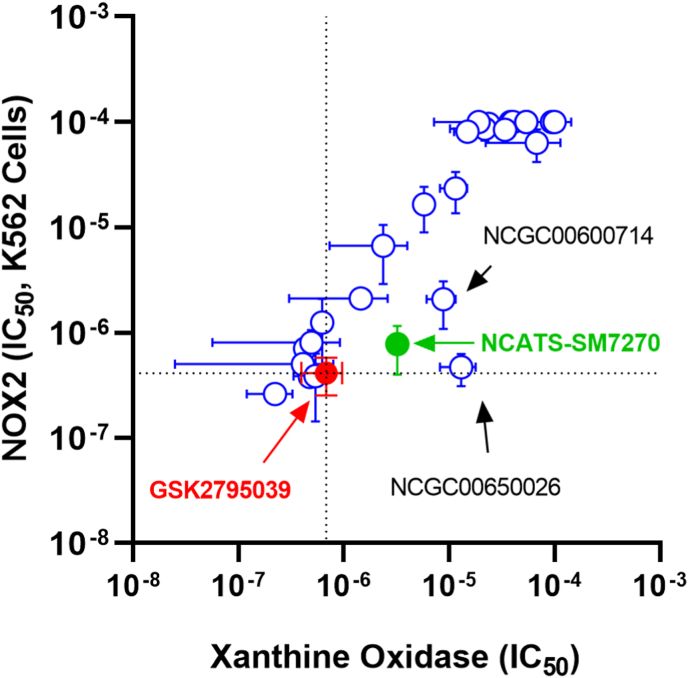
**Inhibitory activity of compounds versus NOX2 and Xanthine Oxidase.** The IC_50_ of compounds were determined in both K562 cells expressing NOX2 and against xanthine oxidase as described. Mean IC_50_ +/− SD are displayed. Dotted lines represent the IC_50_ of GSK2795039 (shown in red) tested in the same assays. NCATS-SM7270 is shown in green and two other promising compounds are indicated. (For interpretation of the references to color in this figure legend, the reader is referred to the Web version of this article.)

GSK2795039 was originally reported to inhibit NOX2 with an IC_50_ of 0.18 μM and required 100x greater concentrations to inhibit xanthine oxidase [26]. However, this compound has a half-life in rodents between 12 min and 2 h [26]. Extensive efforts to identify metabolic byproducts after incubation with liver microsomes revealed several potential modifications to improve stability [28]. A panel of structural variants was prepared (Supplemental Table 1) and their ability to inhibit NOX2 stably expressed in K562 cells and purified xanthine oxidase was measured *in vitro* along with GSK2795039 (Fig. 1). We identified a candidate compound, NCATS-SM7270 (Supplemental Table 1, and methods), with improved permeability, solubility, and half-life in rat microsomes, which retained similar activity against human neutrophil NOX2 and lower activity against xanthine oxidase compared to GSK2795039.

**Fig. 2:**
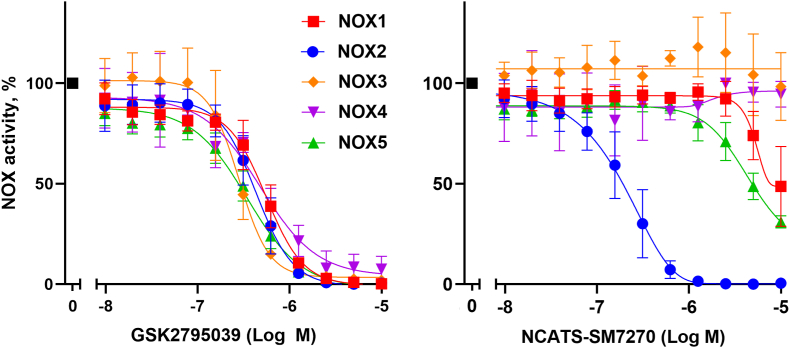
**Dose response curves of GSK2795039 (A) and NCATS-SM7270 (B) in whole cell assays of NOX1-5.** Inhibitory activity of indicated compounds was measured in expi293 cells expressing the indicated NOX family member. Data represent mean±SD for the following number of experiments: 8 (NOX1), 15 (NOX2), 5 (NOX3), 2 (NOX4) and 6 (NOX5).

To further characterize the specificity of NCATS-SM7270 compared to GSK2795039, we developed assays for NOX1-NOX5 that utilized transient overexpression in a serum-free human embryonic kidney cell expression system (Expi293) as described in Materials and Methods. In contrast to the original publication [26], we were unable to demonstrate significant differences in activity of GSK2795039 across the panel of NOX family members (Fig. 2 left panel) although potency against NOX2 was similar to originally reported values. Compared with GSK2795039, NCATS-SM7270 showed a 2-fold improvement in potency toward NOX2, with no detectable activity against NOX3 and NOX4, and only marginal activity at the highest dose tested (about 100x the IC_50_ versus NOX2) against NOX1 and NOX5 (Fig. 2 right panel). The absence of detectable activity of NCATS-SM7270 against NOX3 and NOX4 at doses 100x higher than GSK2795039 provides further evidence that this compound is acting as a NOX2 inhibitor rather than a general ROS scavenger. Another experiment (data not shown) examined the potency of NCATS-SM7270 against NOX1 and NOX3 reconstituted with cytosolic phox proteins (p47phox and p67phox). Here, almost 10-fold higher doses of NCATS-SM7270 were needed to observe inhibition of NOX1 or NOX3 versus NOX2, suggesting this inhibitor targets the core NOX component, rather than affecting assembly of the cytosolic phox regulators. It is worthwhile noting that early studies on GSK2795039 showed its inhibitory potency against NOX2 was diminished by increased NADPH concentrations suggesting its mode of action on NOX2 activity likely involves competitive interactions with the NADPH binding site [26].

When tested against NOX2 in primary human neutrophils, we were unable to detect a significant difference in potency between NCATS-SM7270 and GSK2795039 with IC_50's_ of 4.09 ± 1.00 μM versus 4.87 ± 0.99 μM, respectively (mean of 6 donors ± S. D P > 0.05.). We next evaluated relative potencies in primary mouse neutrophils enriched from bone marrow. Interestingly, in these cells, GSK2795039 (IC_50_ = 2.17 ± 1.11 μM, SD, n = 8 mice) was 2-fold more potent than NCATS-SM7270 (IC_50_ = 4.28 ± 1.05 μM), suggesting that the latter may be slightly less effective at inhibiting NOX2 in mice. Nevertheless, since NCATS-SM7270 was nearly as effective as GSK2795039 in mouse cells and had a more favorable pharmacokinetic and specificity profile, we next tested whether this specific NOX2 inhibitor would show activity in vivo, using a mouse model of mTBI.

### 2.2 The role of NOX family members in mTBI

**Fig. 3:**
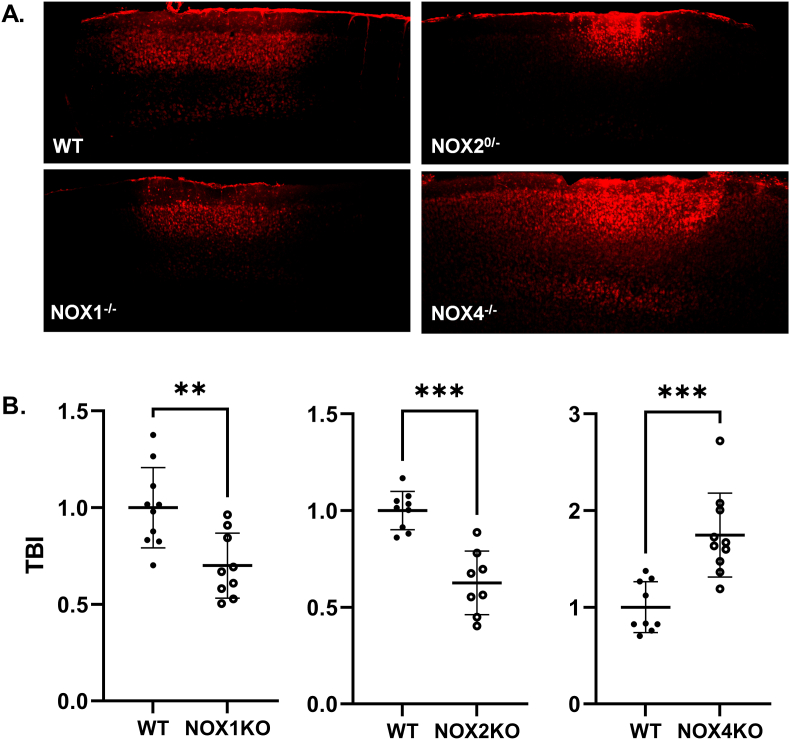
**NOX1-and NOX2-based oxidases facilitate cell death whereas NOX4 ameliorates it after mTBI. (A)** Representative *xy* maximal projection Z stacks (100 μm in depth) show propidium iodide^+^ (PI) dead cells (red) in wildtype (WT), NOX2 KO, NOX1 KO, and NOX4 KO mice. Scale bar: 150 μm. **(B)** Quantification of cell death by selecting only PI^+^DAPI^+^ cells. Each symbol depicts an individual mouse. Bar graphs represent 2 independent pooled experiments with 3–5 mice per group per experiment. Data were normalized to average WT animal per experimental day and displayed as the mean fold change ±SD with **P ≤ 0.01, ***P ≤ 0.001 and ****P ≤ 0.0001 using 2-tailed Student's *t*-test. (For interpretation of the references to color in this figure legend, the reader is referred to the Web version of this article.)

Many studies have addressed the role of NOX2 in TBI and other disease models [20,21,27,[29], [30], [31]]; however, the other NOX isoforms have received less attention. Before testing NCATS-SM7270, we first set out to determine the contribution of three different NOX's (NOX1, NOX2, NOX4) in a single murine mTBI model that involves focal meningeal compression [10,32]. We did not assess the role of NOX3 or NOX5 in this model, because NOX3 expression is limited primarily to the inner ear [33], and NOX5 is not found in rodents [34]. We induced mTBI in wild type as well as NOX1, NOX2, and NOX4 KO mice and quantified cortical cell death 6 h later. Interestingly, both NOX1 and NOX2 deficiency significantly reduced cortical cell death following mTBI, whereas cell death was markedly increased in the absence of NOX4 (Fig. 3). Therefore, while NOX1/2 play a detrimental role in this model of mTBI, the presence of NOX4 is beneficial. These data suggest that broad inhibition of NOX isoforms in the brain may not offer an optimal therapeutic benefit.

**Fig. 4:**
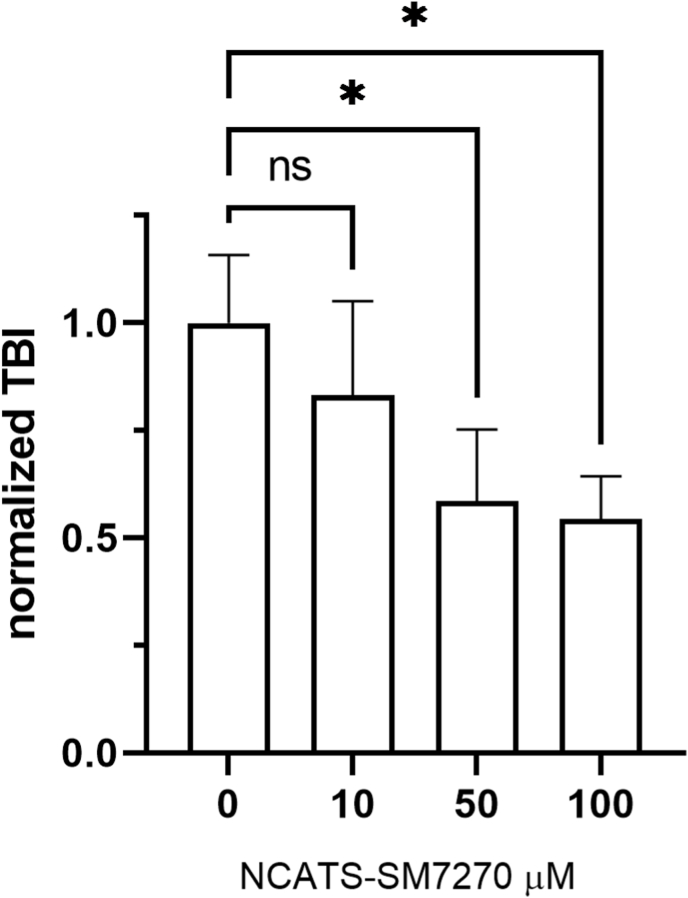
**Dose titration of NCATS-SM7270 in mTBI mice**. Means±SD of 5 mice were treated with vehicle control and 4 mice were treated with either 10, 50, or 100 μM of NCATS-SM7270. Statistically significant (*P ≤ 0.05) reductions in TBI are indicated (Kruskal-Wallis with Dunn's multiple comparison correction).

**Fig. 5:**
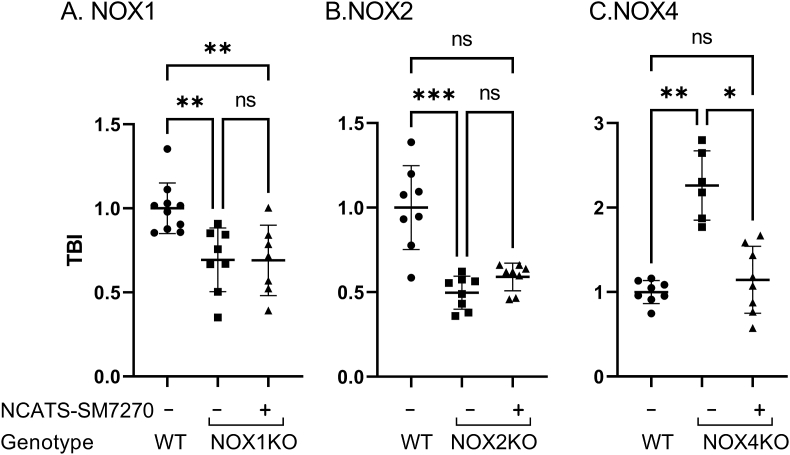
**NCATS-SM7270 is neuroprotective post-mTBI in NOX4 KO but not NOX1 or NOX2 KO mice. (A)** Quantification of cell death in wild type mice or mice deficient in NOX1 (Panel A), NOX2 (Panel B), or NOX4 (Panel C) in the absence or presence of 40 μM NCATS-SM7270. Each symbol represents an individual mouse. All bar graphs represent 2 independent pooled experiments with 3–5 mice per group per experiment. Data were normalized to average DMSO or WT animal per experimental day and displayed as the mean fold change ±SD with *P ≤ 0.05, **P ≤ 0.01, ***P ≤ 0.001, and ns ≥ 0.05 using Kruskal-Wallis with Dunn's multiple comparison test.

To assess the efficacy and off-target effects of our novel NOX2 inhibitor, NCATS-SM7270, we conducted a series of studies in which we prepared compound in artificial cerebrospinal fluid (aCSF) and administered it transcranially immediately following induction of mTBI in mice. This approach offered an opportunity to apply the drug locally to the site of meningeal and brain injury [10]. We first titrated NCATS-SM7270 in our mTBI model using WT mice, and based on efficacy in reducing cortical cell death, selected a concentration of 40 μM, which reduced cell death by ∼50%, for further studies (Fig. 4). We next evaluated the efficiency and selectivity of NCATS-SM7270 by treating NOX1, NOX2, and NOX4 KO mice (Fig. 5). Transcranial administration of NCATS-SM7270 significantly reduced cortical cell death in NOX4 KO mice (Fig. 5C) but had no impact on NOX1 (Fig. 5A) or NOX2 (Fig. 5B) deficient mice. Interestingly, these data suggest that NOX1 and NOX2 function via a similar mechanism to induce cortical cell death, as NCATS-SM7270 did not improve upon the benefit achieved by either deficiency. Our data also indicate that NCATS-SM7270 is a highly effective and specific inhibitor given that it reduced cortical cell death to the level observed in NOX2 knockouts and did not achieve any additional benefit in these mice.

## 3 Discussion

In this study, we describe the development of NCATS-SM7270, a new NOX2 inhibitor with improved specificity, potency, and pharmacologic properties. We also performed a series of experiments to evaluate the role of different NOX isoforms in the same murine mTBI model, revealing that NOX1 and NOX2 play an important role in disease pathogenesis, whereas NOX4 is beneficial. These results differ from a previous study using a different method of inducing TBI showing that NOX4 KO mice are protected from TBI [35]. NOX4 plays a beneficial role in protecting against inflammatory vascular damage in the cardiovascular system [36]. For example, the protective effects of NOX4 have been demonstrated in studies of cardiomyocyte- or endothelial-cell specific NOX4 KO mice [37] although it is still unclear whether a tonic regulatory signal produced by NOX4 acts through transcriptional regulation, e.g., via NRF2 [38] or other mechanisms. Given our mTBI data in different NOX isoform deficient mice, we believe it is very important to treat this injury with a pharmacological inhibitor that has a high degree of specificity for NOX2.

Because NOX2 plays a fundamental role in the pathophysiology of TBI, the development of NCATS-SM7270 is an important step toward the eventual goal of targeting NOX as a therapeutic intervention for humans with brain injuries. We show that NCATS-SM7270 is a highly specific NOX2 inhibitor capable of markedly reducing mTBI-induced cortical cell death after transcranial administration. Importantly, this compound decreased cell death to the same degree as NOX2 deficiency and did not achieve any additional benefit in NOX2 KO mice. These data demonstrate that NCATS-SM7270 is both a specific and efficacious NOX2 inhibitor for proof of concept studies. In future studies, we will evaluate this compound in other pre-clinical disease models and develop additional analogs that maintain specificity for NOX2 or a dual inhibitor of NOX1/NOX2 and exhibit further optimized in vivo pharmacokinetic properties.

## 4 Materials and Methods

### 4.1 Chemical synthesis

All air or moisture-sensitive reactions were performed under a positive pressure of argon with oven-dried glassware. Anhydrous dioxane was purchased from Sigma-Aldrich. Cesium carbonate, K_3_PO_4_, palladium acetate and methanesulfonato[2-(dicyclohexylphosphino)-2'-(*N*,*N*-dimethylamino)-1,1′-biphenyl](2′-amino-1,1′-biphenyl-2-yl)palladium(II) CHCl adduct (DavePhos Palladacycle Gen. 3; CAS # 1445085-87-9) were purchased from Strem Chemicals. Analytical analysis was performed on an Agilent LC/MS (Agilent Technologies, Santa Clara, CA) and both were performed at a flow rate of 1 mL/min. Method 1: A 7-min gradient of 4%–100% acetonitrile (containing 0.025% trifluoroacetic acid, TFA) in water (containing 0.05% TFA) was used with an 8 min run time on a Phenomenex Luna C18 column (3 μm, 3 × 75 mm) at a temperature of 50 °C. Method 2: A 3-min gradient of 4%–100% acetonitrile (containing 0.025% TFA) in water (containing 0.05% TFA) was used with a 4.5 min run time on a Phenomenex Gemini Phenyl column (3 μm, 3 × 100 mm) at a temperature of 50 °C. Purity determination was performed using an Agilent Diode Array Detector for both Method 1 and Method 2. Mass determination was performed using an Agilent 6130 mass spectrometer with electrospray ionization in the positive mode. ^1^H NMR spectra were recorded on Varian 400 MHz spectrometers. Chemical shifts are reported in ppm with undeuterated solvent (DMSO‑*d*_6_ at 2.49 ppm) as an internal standard for DMSO‑*d*_6_ solutions. All compounds tested in the biological assays had a purity greater than 99%, based on both analytical methods. High-resolution mass spectrometry was recorded on Agilent 6210 Time-of-Flight LC/MS system. Confirmation of molecular formula was accomplished using electrospray ionization in the positive mode with the Agilent Masshunter software (version B.02).

### 4.2 Synthesis of NCATS-SM7270

**Figure:**
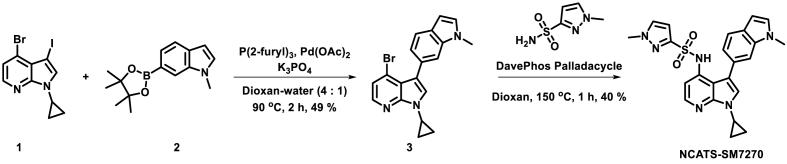

A vial containing a mixture of 4-bromo-1-cyclopropyl-3-iodo-1H-pyrrolo [2,3-b]pyridine **1** (1 g, 2.75 mmol, 1 eq), 1-methyl-6-(4,4,5,5-tetramethyl-1,3,2-dioxaborolan-2-yl)-1H-indole **2** (0.815 g, 3.17 mmol, 1.15 eq), tri-2-furylphosphine (0.064 g, 0.275 mmol, 10 mol %), and potassium phosphate (1.170 g, 5.51 mmol, 2 eq) in 13 mL dioxane/water (4/1 mixture) was bubbled with argon for 5 min then added palladium(II) acetate (0.019 g, 0.083 mmol, 3 mol %). The reaction mixture was capped and stirred at 90 °C for 2 h. The reaction mixture was allowed to cool to room temperature before adding a Pd scavenger resin. The reaction was further stirred for another 30 min, diluted with dichloromethane, and filtered through a Celite pad. The filtrate was concentrated and the residue was directly loaded into a flash silica column. The crude product was purified on a flash system eluting with 0–60% ethyl acetate in hexanes over 32 column volumes using a 25 g silica column to yield 0.496 g (49%) of 4-bromo-1-cyclopropyl-3-(1-methyl-1H-indol-6-yl)-1H-pyrrolo [2,3-b]pyridine **3**. LCMS Retention time: (Method 2) = 3.68 min (M + H) = 366.

An oven-dried microwave vial containing the above product 4-bromo-1-cyclopropyl-3-(1-methyl-1H-indol-6-yl)-1H-pyrrolo [2,3-b]pyridine 3 (0.35 g, 0.956 mmol, 1 eq), Cs_2_CO_3_ (0.623 g, 1.911 mmol, 2 eq) and 1-methyl-1H-pyrazole-3-sulfonamide (0.231 g, 1.433 mmol, 1.5 eq) in dry dioxane was bubbled with argon for 5 min. To the above reaction mixture methanesulfonato[2-(dicyclohexylphosphino)-2'-(*N*,*N*-dimethylamino)-1,1′-biphenyl](2′-amino-1,1′-biphenyl-2-yl)palladium(II) CHCl adduct [39 1445085-87-9] (0.073 g, 0.096 mmol, 10 mol %) was added, capped, and heated in a microwave reactor for 1 h at 150 °C. The reaction was diluted with excess DCM and stirred with a Pd scavenger resin for 1 h. The reaction was filtered through a Celite pad to remove solid suspensions. The filtrate was concentrated and purified on a flash system using a 25 g silica column eluting with 20–100% ethyl acetate in hexanes for 20 column volumes after that at 100% ethyl acetate until the product elutes from the column. The slightly colored solid obtained after evaporating the solvent was suspended in ethanol and allowed to stand overnight at room temperature. The clear solid was collected by filtration. The white solid obtained was dried under a high vacuum to yield a pure product *N*-(1-cyclopropyl-3-(1-methyl-1H-indol-6-yl)-1H-pyrrolo [2,3-b]pyridin-4-yl)-1-methyl-1H-pyrazole-3-sulfonamide (NCATS-SM7270). LC-MS Retention Time: (Method 1) = 7.018 min and (Method 2) = 3.871 min ^1^H NMR (400 MHz, DMSO‑*d*_6_) δ 13.13 (s, 1H), 8.30 (s, 1H), 7.74–7.65 (m, 2H), 7.62–7.52 (m, 4H), 7.47 (td, *J* = 7.7, 0.6 Hz, 1H), 7.24 (dd, *J* = 3.6, 0.5 Hz, 1H), 7.20 (dd, *J* = 11.4, 1.6 Hz, 1H), 7.08 (dd, *J* = 8.2, 1.6 Hz, 1H), 6.83 (dt, *J* = 3.4, 1.0 Hz, 1H), 4.14 (s, 2H), 2.78–2.63 (m, 1H), 2.47 (d, *J* = 1.1 Hz, 3H), 1.80 (ddq, *J* = 75.6, 14.6, 7.6 Hz, 2H), 0.78 (ddt, *J* = 13.1, 8.2, 4.2 Hz, 1H), 0.55 to −0.05 (m, 4H); HRMS (ESI) *m/z* (M + H)^+^ calcd. For C_33_H_28_FN_4_O_4_S_3_ 659.1251, found 659.1282.

### 4.3 Metabolic stability, permeability, and solubility assays

The rat liver microsomal stability [40], parallel artificial membrane permeability assay (PAMPA) [41], and aqueous kinetic solubility assay in phosphate-buffered saline (PBS) buffer [42] were performed as described in the indicated references.

### 4.4 Human cells

Human blood was obtained after informed consent by the Department of Transfusion Medicine (NIH Clinical Center) and provided as de-identified specimens. Human granulocytes were isolated using Percoll density fractionation as described [43] and NOX2 activity was assessed in white polypropylene flat bottom 96 well plates following 15 min of preincubation at 37 °C with compounds or vehicle controls using 50 μM luminol as a chemiluminescence enhancer in an assay volume of 100 μl RPMI1640 with 1.1 × 10^5^ cells/well in Varioskan Lux luminometer for 30 cycles of 1 s per well (approximately 1 h). As published previously, patients with mutations in the gp91 component of NOX2 do not show significant ROS production in this assay [1].

NOX2 activity was also measured using an undifferentiated leukemic cell line, K562, that was stably transfected with gp91phox, p47phox, and p67phox [44]. Cells were assayed after preincubation with inhibitors for 15 min at 37 °C in an assay medium composed of Hank's balanced salt solution without divalent cations containing 10 mM HEPES, 50 μM Luminol, 20 U horseradish peroxidase/mL in a luminometer as above after activation with 100 ng PMA/mL.

### 4.5 In vitro assays for NOX family members

Given concerns about previous assays for specific NOX family members [45] we here report the first use of the Expi293 Expression System (Thermo Scientific) to overexpress NOX1, 2, 3, 4, and 5 in excess of any endogenous NOX proteins present in these cells. Transfections were performed following the manufacturer's instructions using the plasmids for each NOX as described in Supplemental Table 2 except that the enhancer solution provided with the Expi293 system was not used because it promoted enzyme activation.

After the times indicated in Supplemental Table 2, cells were washed and 50,000 added per well of a white 96-well plate (Greiner) along with 50 μM Luminol and 2.5 U horseradish peroxidase/mL in a volume of 90 μl for NOX1, NOX2, and NOX5. Inhibitors or vehicle controls were added and cells were incubated for 20 min at 37 °C prior to activation with the indicated stimuli. For NOX3 and NOX4 (constitutively active), cells were incubated with inhibitor or vehicle control prior to the addition of luminol and HRP. Chemiluminescence was measured every 2 min for 1 s per well at 37 °C in a Varioscan Multimode reader. AUC data were analyzed using GraphPad Prism using non-linear regression analysis after expressing relative to control alone (maximum ROS production).

Bovine Xanthine Oxidase (Sigma) was dialyzed in PBS to remove ammonium sulfate and stored at 4 °C. Hypoxanthine was prepared by heating in DMSO. Reactions were prepared by diluting xanthine oxidase in HBSS (final concentration 1.25 mU/mL) that contained 50 μM luminol and were preincubated 5 min with control or inhibitor before the addition of hypoxanthine at a final concentration of 0.5 mM. Luminescence was measured at 24 °C as described above.

### 4.6 Mice

C57BL/6 J (B6), B6.129 S-Cybbtm1Din/J (NOX2 KO) [46], B6.129X1-Nox1tm1Kkr/J (NOX1 KO) [47], and B6.129-Nox4tm1Kkr/J (NOX4 KO) [48] were purchased from Jackson Laboratories. All mice were maintained in a closed breeding facility and were housed and treated in accordance with the Institutional Animal Care and Use Committee at the National Institutes of Health (NIH). Mice were studied at ages ranging from 7 to 12 weeks and only male mice were used for this study due to the X-chromosomal linkage of the CYBB, the enzymatic center of NOX2.

### 4.7 In vitro mouse studies

Mouse granulocytes were isolated from bone marrow essentially as described [49] except bones were not sterilized in ethanol and marrow was not flushed through a 100 μm strainer. Furthermore, RPMI1640 was supplemented with 10% FBS, 2 mM EDTA, and 25 mM HEPES buffer and penicillin/streptomycin was omitted. Polymorphonuclear leukocytes (PMN) concentration and purity (>90% granulocytes) were assessed microscopically using 3% acetic acid and a hemacytometer. NOX2 activity was assessed as described above with human granulocytes.

### 4.8 In vivo mouse studies

We used a focal cortical model of mTBI to assess the role of NOX family members and NOX2 inhibitors as described previously [9,10,32]. Mice were maintained at a constant temperature of 37 °C during anesthesia with ketamine (85 mg/kg; Ketaset), xylazine (13 mg/kg; AnaSed), and acepromazine (2 mg/kg, VET one) in PBS. NCATS-SM7270 stock solutions were prepared in DMSO and diluted to working concentrations in aCSF (#597316, Harvard Apparatus). Immediately after the mTBI was induced (or as indicated), the inhibitor solution or control aCSF containing equivalent concentrations of DMSO were applied directly to the lesions.

#### 4.8.1 Dead cell detection and quantification

One hour prior to sacrifice (5–6 h post-injury), aCSF with inhibitor or DMSO was washed off the exposed skull, and 200 μL propidium iodide (1 mg/mL, P1304MP, Thermo Fisher Scientific) was applied transcranially to label dead cells as described [9]. Mice then received an intracardiac perfusion with 4% paraformaldehyde (PFA), and heads were incubated overnight at room temperature in 4% PFA. Afterward, brains were sectioned serially with a Compresstome (Precisionary) at a thickness of 100 μm through the entire lesion. A single section with the greatest amount of propidium iodide staining was then imaged through the entire 100 μm of injured tissue using an Olympus FV1200 laser-scanning confocal microscope fitted with a 10 × objective. Imaris version 9.3.1 image analysis software (Bitplane) was used to quantify resultant images. DAPI^+^ “surfaces” were created, and dead cells were counted as DAPI^+^ cells that colocalized with propidium iodide as described [9]. Dead cells were normalized for each experiment by averaging the wildtype or DMSO-treated animal and calculating the fold change for each individual sample relative to the average. Data were then expressed as a fold change.

## Declaration of competing interest

The authors declare that they have no known competing financial interests or personal relationships that could have appeared to influence the work reported in this paper.

## Data Availability

Data will be made available on request.
