# Supplementary material for: Development of an improved and specific inhibitor of NADPH oxidase 2 to treat traumatic brain injury
Source: Redox Biol. 2023 Jan 18;60:102611. doi: 10.1016/j.redox.2023.102611 (PMC9894920; doi:10.1016/j.redox.2023.102611)
Supplement: Multimedia component 2 [file mmc2.docx]

Supplemental Table 2

| NADPH oxidase isoform | NCBI Reference Sequence  For NOX component | Plasmids | Time for protein expression | Compounds used for activation of ROS production |
| --- | --- | --- | --- | --- |
| NOX1 [1] | NM_007052  NM_172167  NM_006647  NM_000101  NM_006908 | pcDNA3.4-TOPO-NOX1  pcDNA3.4-TOPO-NOXO1b  pcDNA3.4-TOPO-NOXA1  pcDNA3.4-TOPO-p22-phox  pcDNA3.4-TOPO-Rac1 | 24 hours | PMA, 100 ng/ml |
| NOX2 [1] | NM_000397  NM_000265  NM_000433  NM_000101  NM_006908 | pcDNA3.4-TOPO-NOX2  pcDNA3.4-TOPO-p47-phox  pcDNA3.4-TOPO-p67-phox  pcDNA3.4-TOPO-p22-phox  pcDNA3.4-TOPO-Rac1 | 24 hours | PMA, 100 ng/ml |
| NOX3 [1] | NM_015718  NM_172167  NM_000101 | pcDNA3.4-TOPO-NOX3  pcDNA3.4-TOPO-NOXO1b  pcDNA3.4-TOPO-p22-phox | 24 hours | None |
| NOX4 [2] | AF261943  NM_000101 | pcDNA3.4-TOPO-NOX4  pcDNA3.4-TOPO-p22-phox | 48 hours | None |
| NOX5 [3] | NM_001184779 | pcDNA3.4-TOPO-NOX5b | 24 hours | Ionomycin, 1 µM  PMA, 100 ng/ml |

References

1. Ueyama, T., M. Geiszt, and T.L. Leto, *Involvement of Rac1 in activation of multicomponent Nox1- and Nox3-based NADPH oxidases.* Mol Cell Biol, 2006. **26**(6): p. 2160-74.

2. Geiszt, M., et al., *Identification of renox, an NAD(P)H oxidase in kidney.* Proc Natl Acad Sci U S A, 2000. **97**(14): p. 8010-4.

3. Banfi, B., et al., *A Ca(2+)-activated NADPH oxidase in testis, spleen, and lymph nodes.* J Biol Chem, 2001. **276**(40): p. 37594-601.
